# Editorial: Sport and psychosocial health/well-being after the COVID-19 lockdown - Volume II

**DOI:** 10.3389/fspor.2023.1256235

**Published:** 2023-07-24

**Authors:** Amy Chan Hyung Kim, James Du, Rochelle Eime

**Affiliations:** ^1^ Center for Sport, Health, and Equitable Development, Department of Sport Management, Florida State University, Tallahassee, FL, United States; ^2^ Institute for Health and Sport, Victoria University, Melbourne, VIC, Australia; ^3^ Physical Activity and Sport Insights, Research and Innovation, Federation University, Ballarat, VIC, Australia

**Keywords:** sport, physical activity, exercise, mental health, psychological well being, psychosocial health and well-being, COVID- 19

**Editorial on the Research Topic** Sport and psychosocial health/well-being after the COVID-19 lockdown - Volume II

Since 2019, the COVID-19 pandemic has made a significant impact on one's participation patterns in physical activity, exercise, and sport due to a range of restrictions all across the world including extensive stay-at-home orders and the cancellation of sport training and competitions.

The first volume of this Research Topic on Sport and Psychosocial Health/Well-being after the COVID-19 Lockdown was launched in 2020 and published a total of 11 articles. These studies included a wide range of different physical activities across the spectrum form organised competitive sport to nature-based outdoor activities. Further the studies included various populations such as general population, elite athletes, Paralympic athletes, patients with health conditions, and sport volunteers. In this new volume II, a total of 6 original research articles were published which also covers various context of sport and physical activity with heterogenous populations including adolescents, adults, recreational golfers, elite college soccer players, and patients with prolonged symptoms after COVID-19 infection. The nationality of samples included France, Germany, Austria, Canada, China, and South Korea.

In this second volume, three articles examined the important role of physical activity, exercise and sport on one's mental health during the pandemic. Colas et al. assessed the effectiveness of Telerehabilitation program called CoviMouv fatigue among patients with prolonged symptoms after COVID-19 infection. This program is a mixed remote program that offers adapted physical activity programs and therapeutic education. After the program, the results showed that fatigue was reduced while aerobic performance was improved, revealing that remotely designed adapted physical activity and education programs can be helpful for this population group.

Similarly, but by using a nonexperimental design, Hu et al. examined the relationship between home-based High-Intensity Interval Training (HIIT) dance and depression during the COVID-19 pandemic in China while examining the moderating effects of personal perception including perceived susceptibility, severity, benefits, and self-efficacy among quarantined residents in China. The findings showed that more often the quarantined residents participated in home-based HIIT dance, the less likely they were to suffer from depression during the lockdown. While perceived susceptivity did not play a significant role as a moderator on this preventive relationship between HIIT dance frequency and depression, higher perceived severity and perceived benefits tended to strengthen the association. Moreover, if the quarantine residents had a clear sense that they could effectively participate in home-based HIIT dance, it inclined to prevent their experience of depression more greatly.

Jungwirth et al. examined the impact of the COVID-19 crisis on individual physical activity patterns and life satisfaction among recreational golfers. The participants recruited from German speaking countries reported that, in indoor settings, the frequency of fitness center use was decreased by 42.12% while the frequency of home training was increased by 14.18% (independent training) and 23.48% (with online instructions). Notably, life satisfaction had decreased significantly after the COVID-19 pandemic among the respondents compared to before the COVID-19 pandemic.

The remaining three articles explored the different patterns or situational factors of physical activity behavior during the COVID-19 pandemic. Kim et al. examined how the degree of conflict (i.e., conflict due to prejudice, conflict due to competition, conflict due to prior expectations, conflict due to not observing etiquette) and coping strategies (i.e., avoidance behavior, resolution behavior) varies depending on spatial proximity that occurs in indoor and outdoor settings. The results from 508 Korean adults disclosed that conflict due to prejudice was higher in indoor sport activities such as Pilates, yoga, and gym workouts. Conflicts due to competition were relatively low in these activities though in that these activities were only available via reservation or appointments in order to limit the number of people within a certain size of indoor spaces during the pandemic in South Korea. The results also indicated that indoor golf showed higher level of conflict from competition and conflict due to not observing etiquette in indoor golf settings compared to outdoor golf settings. Outdoor activities such as jogging and hiking showed higher conflicts due to prior expectations and prejudice. The results implied that, during the time under nonpharmaceutical restrictions, physical activity participants may feel different conflicts and adopt coping strategies depending on the type of activities and environmental contexts. The policymakers and leisure service providers need to consider different conflicts and coping strategies among leisure participants when they run different programs under various restrictions that may resulted from major events such as the COVID-19 pandemic.

Kovacevic et al. investigated the changes in adolescents’ physical activity behaviors and cognitions associated with COVID-19 using the Multi-Process Action Control (M-PAC) framework. The findings from a total of 588 grade 11 students in Ontario, Canada presented that participants were 67% less likely to participate in organized physical activities during the pandemic compared to before the pandemic. When it comes to physical activity cognitions, physical activity intention, identity, and habit significantly decreased over time while behavioral regulation was increased. Considering that many restrictions result in disruptions to typical behaviors, adolescents had to implement new skills and plans to be physically active. The findings implied the need of more support on helping adolescents regulate their behaviors through various skills such as self-monitoring, goal setting, and coping strategies.

Lastly, Zhao et al. explored the effect of perceived social support on mental health among college soccer players in China while investigating the mediating effect of burnout and hopelessness during the COVID-19 lockdown (February 2022). The findings from 695 research participants in China indicated that the higher perceived social support was associated with lower level of psychological distress while athlete burnout and hopelessness mediated the relationship between perceived social support and psychological distress.

In the end, volume II extended knowledge in how physical activity, exercise, and sport play roles in one's mental health during the COVID-19 pandemic as well as how one's active lifestyle has been affected by the recent pandemic. In summary the pandemic impacted individuals’ sport and physical activity behaviors which in turn negatively impacted a range of health outcomes. The crucial message from this issue is that one's active lifestyle should be promoted effectively to cope one's mental health issues that can be resulted from the COVID-19 pandemic. Also, as the pandemic restrictions are lifted and we have the opportunity to return to playing sport and being physically active, the impact of the pandemic and associated restrictions are likely to continue to impact our sport and physical activity behaviors. This in turn will impact individuals’ health and wellbeing and the long-term effects of the COVID-19 on one's active lifestyle should continue to be examined.

